# Supplementary figures and images for: Chromatin endogenous cleavage provides a global view of yeast RNA polymerase II transcription kinetics
Source: eLife. 2024 Nov 28;13:RP100764. doi: 10.7554/eLife.100764 (PMC11604220; doi:10.7554/eLife.100764)

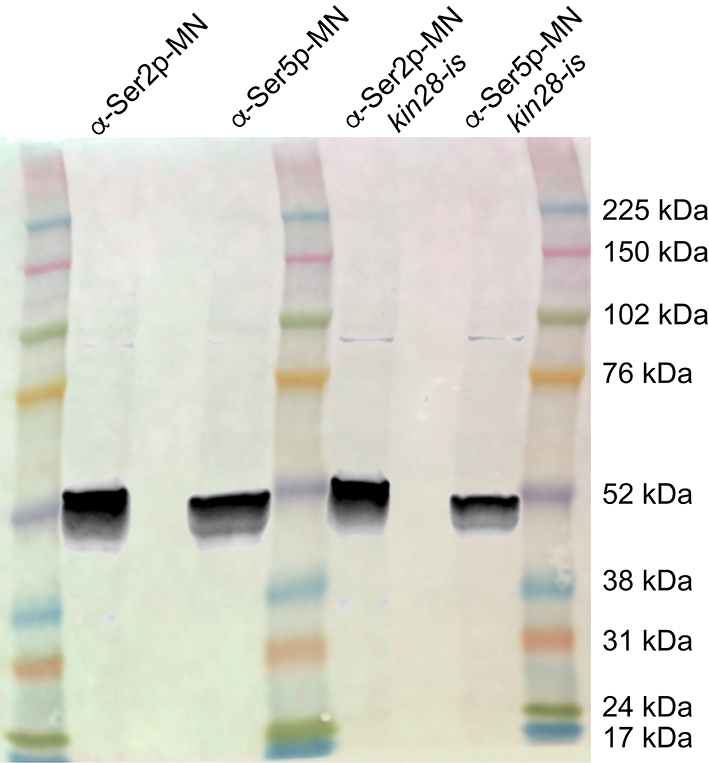

Supplement: Figure 3—figure supplement 1—source data 1. [file elife-100764-fig3-figsupp1-data1.zip › 8fdf0a65-1a3e-440b-baea-0b34473759ff.tif]

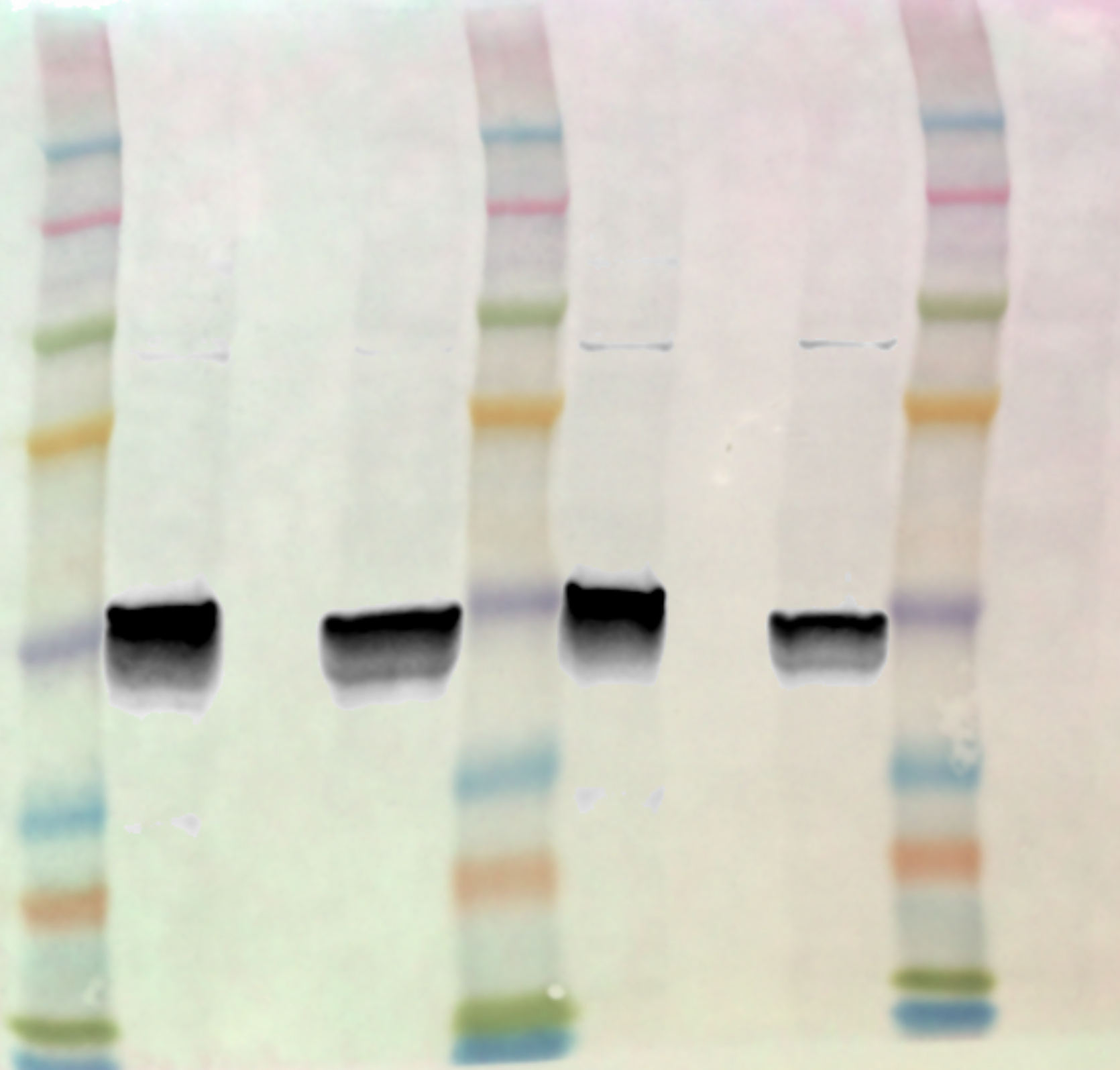

Supplement: Figure 3—figure supplement 1—source data 2. [file elife-100764-fig3-figsupp1-data2.zip › Figure 3 marker blot.tif]

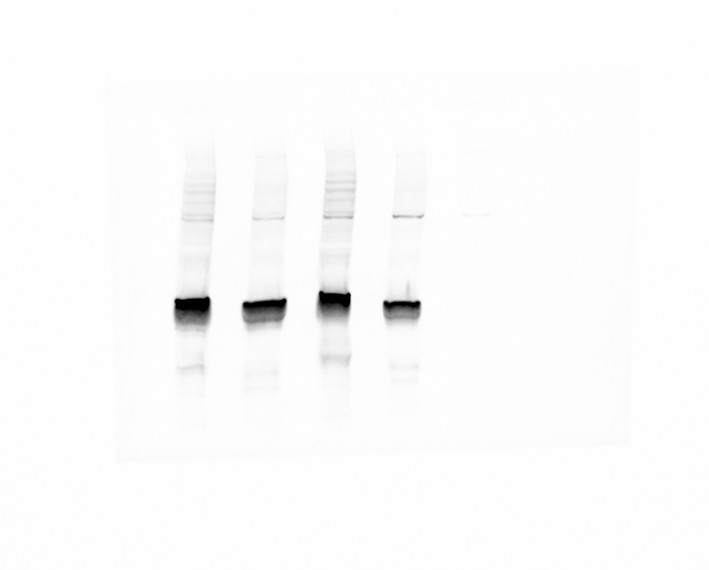

Supplement: Figure 3—figure supplement 1—source data 2. [file elife-100764-fig3-figsupp1-data2.zip › Figure 3 - figure supplement 1 blot.tif]

Biological Replicate:           #1           #2           #3  
Duration 3-IAA (min):       0 10 20 30   0 10 20 30   0 10 20 30

Sua7-3xV5-IAA7

Actin

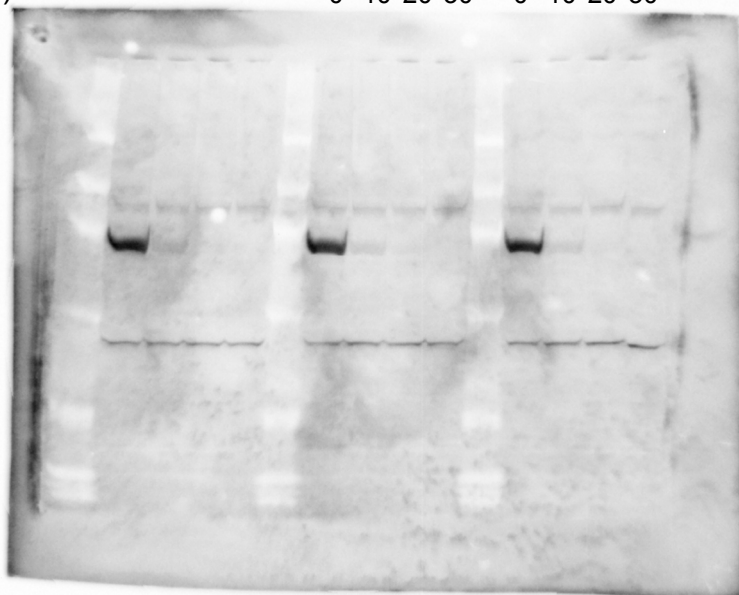

Supplement: Figure 5—source data 1. — Three biological replicates are shown. Samples were collected 0, 10, 20, and 30 min following the addition of 3-IAA. Rainbow molecular weight marker is loaded in the lane immediately to the left of each 0 min time point. Biological replicate #1 is shown in Figure 5A. [file elife-100764-fig5-data1.zip › Figure 5-source data 1/Figure 5 Source Data 1.pdf]

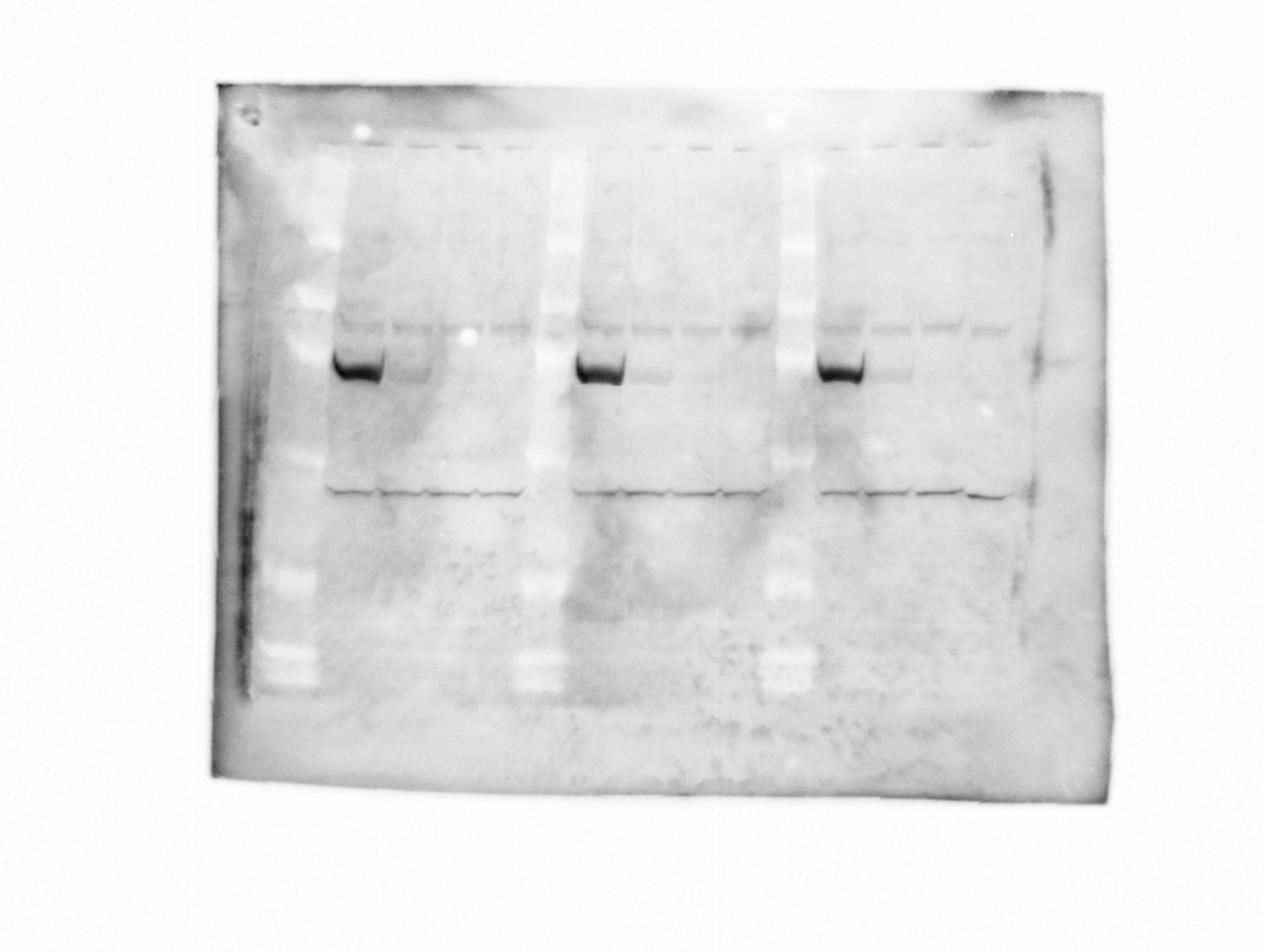

Supplement: Figure 5—source data 2. [file elife-100764-fig5-data2.zip › Figure 5-source data 2/Western Blot Sua7-3V5-IA77 auxin time course.tif]
